# Polypropylene as a Retrofitting Material for Shear Walls

**DOI:** 10.3390/ma13112503

**Published:** 2020-05-30

**Authors:** Enea Mustafaraj, Yavuz Yardim, Marco Corradi, Antonio Borri

**Affiliations:** 1 Department of Civil Engineering, EPOKA University, 1039 Tirana, Albania; emustafaraj@epoka.edu.al; 2 Department of Civil and Environmental Engineering, Edinburgh University, Edinburgh EH9 3FG, UK; yyardim@exseed.ed.ac.uk; 3 Department of Mechanical & Construction Engineering, Northumbria University, Newcastle upon Tyne NE1 8ST, UK; 4 Department of Engineering, University of Perugia, 06125 Perugia, Italy; antonio.borri@unipg.it

**Keywords:** polypropylene fibers, polypropylene net, earthquake engineering, masonry retrofitting methods, masonry structures, mechanical testing

## Abstract

In recent years, on account of their excellent mechanical properties, composite materials (made of epoxy-bonded carbon, glass, or aramid fibers) have been used to reinforce masonry walls against in-plane actions. These materials have proven to be an effective solution for the strengthening of unreinforced masonry (URM) walls. Lately, research has shifted to the study of different types of fibers to avoid the use of epoxy adhesives, whose long-term behavior and compatibility with masonry are poor. This paper describes an experimental program that investigated the behavior of URM shear walls strengthened with two types of commercially available polypropylene products: short fibers (fiber length = 12 mm) and polypropylene nets. This investigation aimed to evaluate the influence of polypropylene reinforcement, embedded into an inorganic matrix, in terms of the improvement of the lateral load-carrying capacity, failure mechanism, ductility, and energy dissipation capacity of URM wall panels, where nine walls were subjected to in-plane loads using a racking test setup. The study showed that using two layers of polypropylene fibers embedded into a cementitious matrix greatly increased the in-plane load capacity of the brickwork masonry. On the other hand, the test results indicated that polypropylene nets, used as a repair method for cracked shear walls, cannot improve the structural performance of the walls.

## 1. Introduction

Fiber-reinforced polymers (FRP) have been gaining widespread use in the past few years, not only in civil infrastructure but also in conservation engineering. Their high tensile strength-to-weight ratio and durability make them ideal for the repair and retrofitting of infrastructures and historic buildings.

FRP reinforcement is often necessary to increase the capacity of historic constructions against natural hazards. Natural disasters, such as earthquakes, floods, and tsunamis, can damage the building stock with destructive effects. Earthquakes are a serious problem for historic constructions: south-east Europe is particularly exposed to the effects of earthquakes, with a high level of seismic activity in many countries (Albania, Greece, Italy, Montenegro, Slovenia, etc.). It is well known that historic masonry buildings are mainly affected by the horizontal forces created by the earthquakes [1,2,3,4,5,6].

Depending on the direction of the seismic action, masonry walls may be subjected to in-plane or out-of-plane loading. The wall instability, called out-of-plane rocking, is the most dangerous failure mode of historic buildings [7,8]. This out-of-plane behavior is typically dominated by the equilibrium conditions of the single-wall panels, regardless of the mechanical properties of the masonry material. An effective method used to avoid rocking walls is to increase the effectiveness of wall-to-wall connections. This can be achieved using different methods: ring beams [9], application of local reinforcements at walls intersections [10], installation of steel ties [11,12], etc.

However, the ultimate load capacity of masonry walls subjected to in-plane loads is typically governed by material failures. Historic buildings in Europe are often made of brickwork or ashlar stonework masonry; apart from the rare use of very soft stone (tuff, sandstone, etc.) or earth bricks, the mechanical properties of historic masonry depend on the type of mortar used, which is often a lime with low mechanical properties. The tensile and compressive strengths of the mortar have a critical influence on the in-plane load capacity.

**Figure 1:**
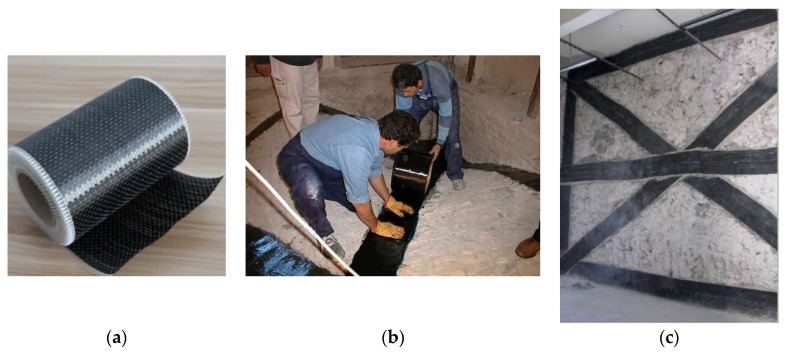
Application of a carbon-fiber-reinforced polymer (CFRP) sheet: (**a**) a CFRP bi-directional sheet, (**b**) use of epoxy-resins to bond the CFRP on the masonry surface, and (**c**) a reinforced wall panel.

Figure 1a shows a bi-directional carbon-fiber-reinforced polymer (CFRP) sheet. This material was the first to become popular in civil engineering in the 1990s to reinforce deficient reinforced concrete (RC) beams (direct application on the tension side) or RC columns (by wrapping). Its application is relatively straightforward: RC surfaces, following an accurate cleaning through the removal of any inconsistent material, are impregnated with an epoxy resin, which is used to glue the carbon fibers to the concrete material. In conservation work, CFRP sheets were initially used to reinforce masonry vaults (Figure 1b) and walls (Figure 1c) against the lateral loading induced by earthquakes [13,14,15,16]. 

**Figure 2:**
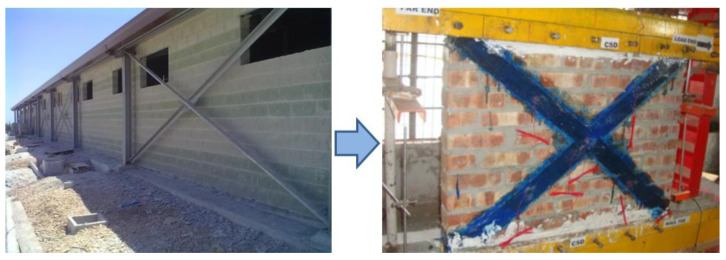
Statics of the resisting mechanism: from a steel structure to a CFRP-reinforced wall [13].

The in-plane reinforcement of wall panels aims to reproduce the concept of X and V steel bracings in metal structures (Figure 2). FRP sheets have the function of absorbing tensile stresses and transferring them to the foundations of the masonry structure. Masonry, similarly to concrete, exhibits a very low tensile strength and a high compressive strength. The low tensile strength also affects both the bending and shear strengths. These are typically very low for masonry. Because earthquakes produce tensile stresses in masonry, FRP reinforcement has been proposed as a retrofitting method. Its effectiveness highly depends on the bonding between the masonry material and the composite fibers, where the basic idea is to create a new “composite material” such that compressive stresses are absorbed by masonry and tensile ones by the FRP. A very large number of laboratory experiments [17,18], on-site applications [19,20], and theoretical studies [21,22] have been conducted in the last two decades using epoxy-bonded fibers.

Due to the development of new retrofitting methods for historic constructions, the need arose to develop a means of avoiding the use of epoxy adhesives. In fact, at the beginning of the 2010s, doubts and concerns were raised about the long-term behavior of the epoxy adhesives used in civil engineering. It has been demonstrated that the bonding properties of epoxies tend to deteriorate with exposure to light, humid environments, and high temperatures [23,24], thus compromising the reinforcement action of the fibers. A further consideration advising prudence in the use of epoxy-bonded reinforcements was raised by conservators: epoxies may permanently damage the masonry material since they irreversibly penetrate the porous stones and bricks.

**Figure 3:**
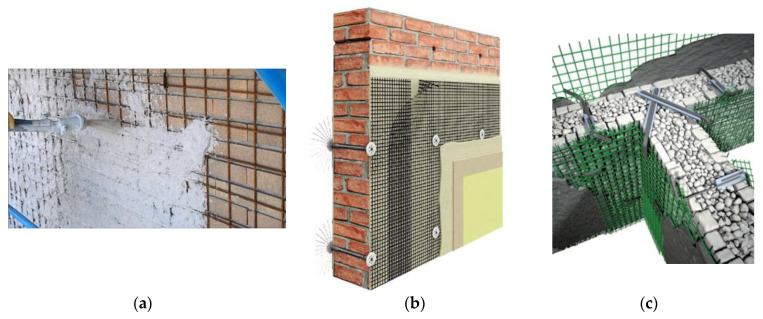
Traditional and innovative methods for the in-plane reinforcement of load-bearing walls: (**a**) steel-rebar-reinforced concrete jacket, (**b**) Fiber Reinforced Matrix (FRM)—flexible non-impregnated fiber meshes, and (**c**) FRM—impregnated (rigid) glass-fiber-reinforced polymer (GFRP) mesh embedded into a cementitious/lime matrix [30,31].

**Figure 4:**
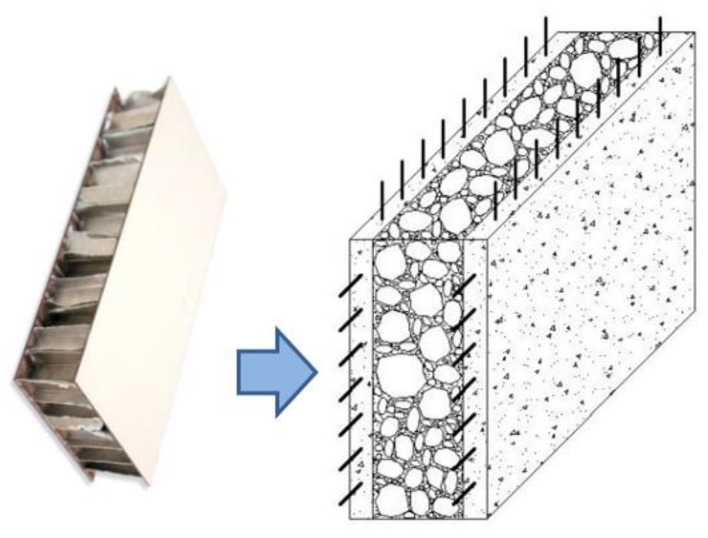
Statics of the resisting mechanism: from a sandwich panel to an FRM-reinforced wall.

The proposed solution was to apply FRP reinforcement using non-polymeric matrices; textile reinforced mortars (TRMs) have recently been studied and tested as an in-plane reinforcement of wall panels (Figure 3) [25,26,27,28,29]. The results demonstrated that it is possible to increase the masonry shear strength using FRP meshes embedded into a cementitious mortar or a plaster. The bonding between the masonry and the reinforcement is significantly weaker when non-polymeric matrices are used, but by increasing the bonded surface, it is possible to reduce the bonding stresses at the interface. The result is that entire wall faces are typically reinforced with TRMs. By doing this, there is a radical shift from the concept of X and V bracings that are typically adopted for epoxy-bonded FRP reinforcements to the scheme of a sandwich structure, where the two thin and stiff skins are made with the TRM and the thick core is the masonry (Figure 4). 

In this paper, the concept of a sandwich structure has been used. Short polypropylene fibers were employed to reinforce brickwork wall panels. Polypropylene fibers were embedded into a cementitious mortar and applied by hand using a standard trowel. Two thin layers of reinforced mortar were laid on both wall faces. The wall panels were tested in shear before and after the application of the polypropylene reinforcement. 

## 2. Polypropylene in Conservation Engineering

In this experimental work, two types of products made of polypropylene were studied and used for the shear reinforcement of wall panels: polypropylene fibers and a polypropylene net.

Polypropylene is a synthetic material that is made of a thermoplastic polymer and produced using a chain-growth polymerization from monomer propylene. Nowadays, polypropylene is the second-most extensively manufactured commodity plastic (after polyethylene) and it is very often employed for labeling and packaging [32,33].

In civil engineering, polypropylene fibers became popular in the late 1990s for their ability to reinforce concrete members given they are very cost-competitive, lightweight, and can be added without difficulty in the cement mix. In contrast, applications on masonry members are rare, and research in this area is very fragmented and scattered. However, polypropylene fibers exhibit very remarkable properties, making their use of interest for the seismic upgrading interventions of masonry buildings. Unlike steel bars and fibers, these fibers are chemically inert, relatively inexpensive, and their low Young’s modulus can produce an increase in the elasticity and load-carrying potential of reinforced masonry members.

These fibers are typically produced as continuous cylindrical filaments that are cut to a specified length. A very important parameter to consider is the design volume of polypropylene fibers: for structural applications, research has demonstrated that design volumes greater than 2% can reduce or control drying shrinkage and improve the ultimate strength of reinforced concrete [34].

However, a large number of building codes and international standards restrict the engineering basis for polypropylene fiber reinforcement to mostly non-structural applications, which are only used for reducing the drying shrinkage and cracking of cement-based mixes [35,36].

The main shortcoming of polypropylene fibers is the low fiber-to-matrix bonding. The low value of Young’s modulus (typically between 1300 and 1500 MPa) can also be considered a problem when the aim is to transfer the load from the masonry to the polypropylene reinforcement. In the conservation engineering of masonry constructions, several advantages come from the use of polypropylene fibers: these fibers are typically added to the mortar during mixing, thus eliminating reinforcement placing operations and their associated costs, and polypropylene is resistant to almost all organic solvents and moisture. Furthermore, it has been demonstrated that the inclusion of polypropylene fibers increases the impact resistance of concrete; this property is of interest in earthquake engineering given the similarities between the seismic action and impact loading. 

**Figure 5:**
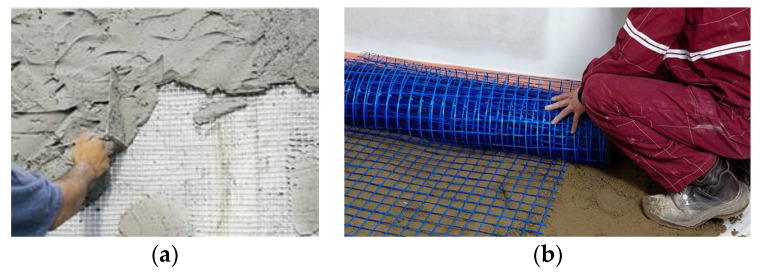
Non-structural applications of polypropylene nets: (**a**) repairing of deteriorated plasters and (**b**) stabilizing the screed before the application of the floor finish.

Propylene was first polymerized to a crystalline isotactic in 1954, which led to large-scale commercial production of isotactic polypropylene by the Italian firm Montecatini from 1957 onwards. Polypropylene nets and meshes became popular in the 1970s for many structural applications, such as sheets of yacht sails, fishing and agricultural nets, and safety fencing. In civil engineering, the applications are typically non-structural, where polypropylene nets are often used for the repair of deteriorated plasters and floors in old buildings (Figure 5) [37,38,39,40].

**Figure 6:**
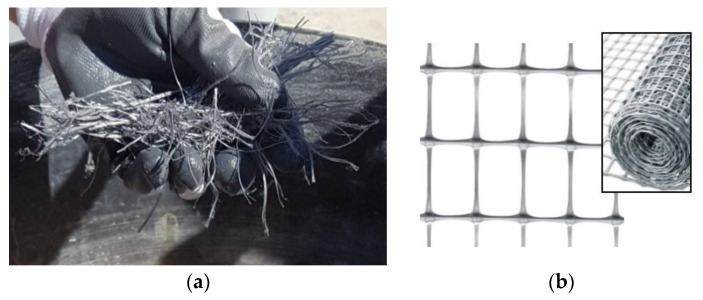
(**a**) Polypropylene fibers used for the panel reinforcement. (**b**) Polypropylene netting used for the panel reinforcement (spacing 30 mm × 45 mm).

In this experimental work, polypropylene fibers (PF) with a circular cross-section (Figure 6a) and polypropylene nets (PNs) (Figure 6b) were studied and tested as a structural method for reinforcing the shear wall of old buildings. 

Table 1 and Table 2 show the main mechanical properties of the polypropylene fibers and nets used for the masonry reinforcement. 

The full test matrix is shown in Table 3. Each shear test was identified with an alphanumeric label: the letter designations SW, W, and LW were used to identify the dimensions of the tested wall panels; each panel was labeled with a number (from 1 to 9, for a total of nine wall panels of different dimensions); PF (polypropylene fibers) and PN (polypropylene net) identify the reinforced panels, while the letter designation URM was used for unreinforced masonry. All wall panels, except panel nos. 7 and 8, were made of solid bricks. These two panels were constructed using ruble (barely cut) calcareous stones.

## 3. Tests Using Polypropylene Fibers

The first part of the experimental study examined the structural performance of the URM wall panels that were retrofitted using a cementitious PF-reinforced mortar. Two types of wall panels were tested in shear: full-scale specimens (W-series) and small-scale specimens (SW-series).

The solid clay bricks that were used were obtained from a kiln located in Fier, Albania. For the construction of the full-scale and small-wall specimens, bricks of two dimensions were used (full-dimension and half-size bricks): both types of bricks were manufactured using identical constituent materials at the kiln.

### 3.1. Masonry

**Figure 7:**
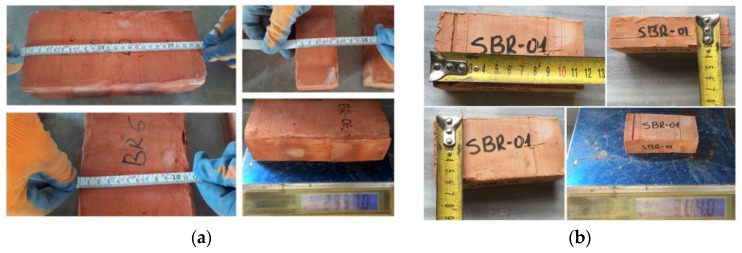
Solid fired clay bricks used for the construction: (**a**) full-dimension and (**b**) half-size.

Both types of solid bricks were made of fired clay. The average full-dimension brick (BR) size was 244 mm × 116 mm × 56.5 mm with a weight of 2778 g on average. The half-size brick (sBR) was 119 mm × 58 mm × 30.6 mm and weighed 372 g on average (Figure 7). The two types of brick were tested in compression and in tension: the mechanical properties of BR were found to be about 20% higher compared to sBR. The compressive and tensile strengths of BR bricks were 24.3 and 4.53 MPa, respectively (Table 4). The coefficient of variation of these results ranged between 10% and 16%.

The mortar used for the construction was cementitious. The cement and the lime were obtained from Fushe Kruje Cement Factory LLC, Kruje, Albania. The grade of the used cement was 32.5 (marked CEM II/B-L 32.5 R), which is suitable due to its lower water demand and improved workability. The mortar mix used for the panel construction was made of hydraulic cement mortar with a volumetric cement:lime:sand ratio of 1:2:9. Mortar prisms were tested following the American Standards for Testing and Materials (ASTM) C109 procedure [41]. The average compressive and tensile strengths were 3.02 and 1.97 MPa, respectively.

**Figure 8:**
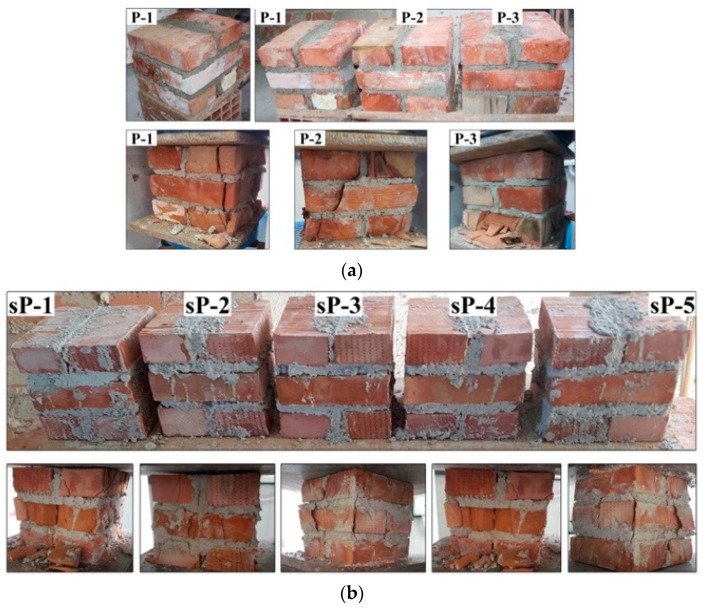
Masonry prisms tested in compression (before and after testing): (**a**) full-scale brick and (**b**) half-scale brick.

The masonry compressive strength was determined by compression testing small prisms made of the same bricks and mortar mix as the panels. The masonry prisms made of full-dimension bricks are shown in Figure 8a, where the prisms are shown before and after the test. As seen in Table 2, the average compressive strength for masonry prisms is 8.36 MPa. On the other hand, the compressive strength of the prisms made of half-scale bricks was 13.69 MPa (Figure 8b). 

For the mortar used to plaster the wall panels, the compressive strength and the flexural strengths were 17.64 and 2.12 MPa, respectively.

### 3.2. Test Method

**Figure 9:**
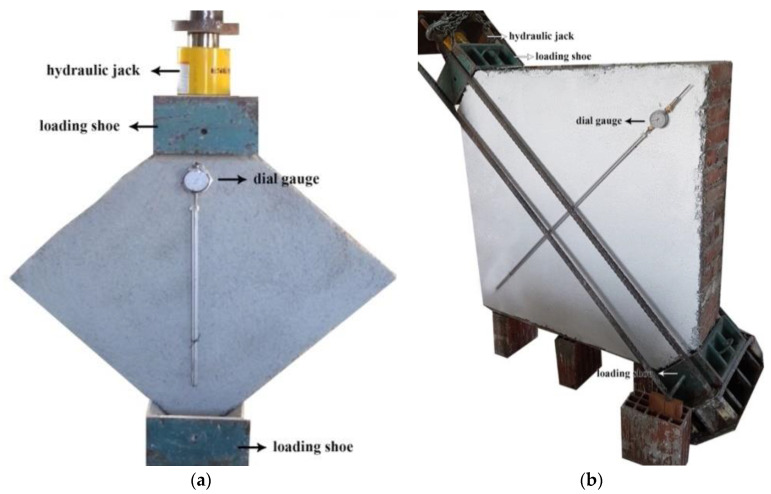
Diagonal compression test setup: (**a**) half-size wall panel and (**b**) full-size wall panel.

The determination of the diagonal tensile strength (shear strength) was done according to the ASTM E 519-02 standard [42]. According to this standard, it is required that the panel is rotated by 45° and a vertical compressive load is applied along the panel diagonal. For the small wall panels, the test setup consisted of two steel loading shoes placed at the top and bottom corners of the rotated panel, along with a 50-tonne-capacity hydraulic jack. On both sides, two dial gauges were used to measure the shortenings and elongations of the panel under shear loading (Figure 9a).

For the full-scale panels, to avoid damaging or disturbing the overall conditions of the wall, the brickwork specimens were tested in place, whereas the loading mechanism was rotated, as shown in Figure 9b. The test setup was made of two loading steel shoes placed at the top and bottom edges of the loaded wall diagonal, where two steel profiles that were mutually connected by four high-strength steel ties, used to contrast the diagonal load. This was applied using a 50-tonne-capacity hydraulic jack, which was placed between a steel profile and a shoe. The jack was connected to a hydraulic pump and a one-way valve permitted oil to flow due to pressure from the pump to the jack and to lift the load, compressing the wall diagonally. The load was increased gradually until failure. As in the small panels, the deformations were recorded using two dial gauges positioned on both sides.

The calculation of the mechanical parameters was done using the following equations:

(1) $$S_{S}=0.707\frac{P}{A_{n}},$$

where *S_S_*—shear stress (MPa), *P*—load exerted along the compression diagonal (N), and *A_n_*—net area of specimen (mm^2^).

(2) $$A_{n}=\frac{w+h}{2}\left(t\times n\right),$$

where *w*—width of the specimen (mm); *h*—height of the specimen (mm); *t*—total thickness of the specimen (mm); and *n*—percent of the gross area of the unit that is solid, expressed as a decimal.

(3) $$\gamma =\frac{\Delta V+\Delta H}{g},$$

where *γ*—shearing strain (mm/mm), ∆*V*—vertical shortening (mm), ∆*H*—horizontal extension (mm), and *g*—vertical gage length (mm).

(4) $$G=\frac{S_{S}}{\gamma },$$

where *G*—modulus of rigidity (shear modulus) (MPa).

Since masonry is a heterogeneous material and the behavior does not have a distinct yielding point, the shear modulus can be determined using the slope of a secant line (secant modulus) on a stress–strain plot; for this study, two shear moduli, *G*_1_ and *G*_2_, were calculated. *G*_1_ was calculated using two fixed values of the shear load (half-scale panels 0–30 kN, full-scale panels 0–50 kN), while for *G*_2_, the values of 0.05*S_S_* and 0.70*S_S_* (5% and 70% of the shear strength) of the maximum shear stress and the corresponding strain were adopted. The Young’s modulus, *E*, can be related to the shear modulus by using Equation (5), where *ν* = 0.25 is the Poisson ratio:

(5) E=2G(1+ν).

### 3.3. Test Results and Discussion

**Figure 10:**
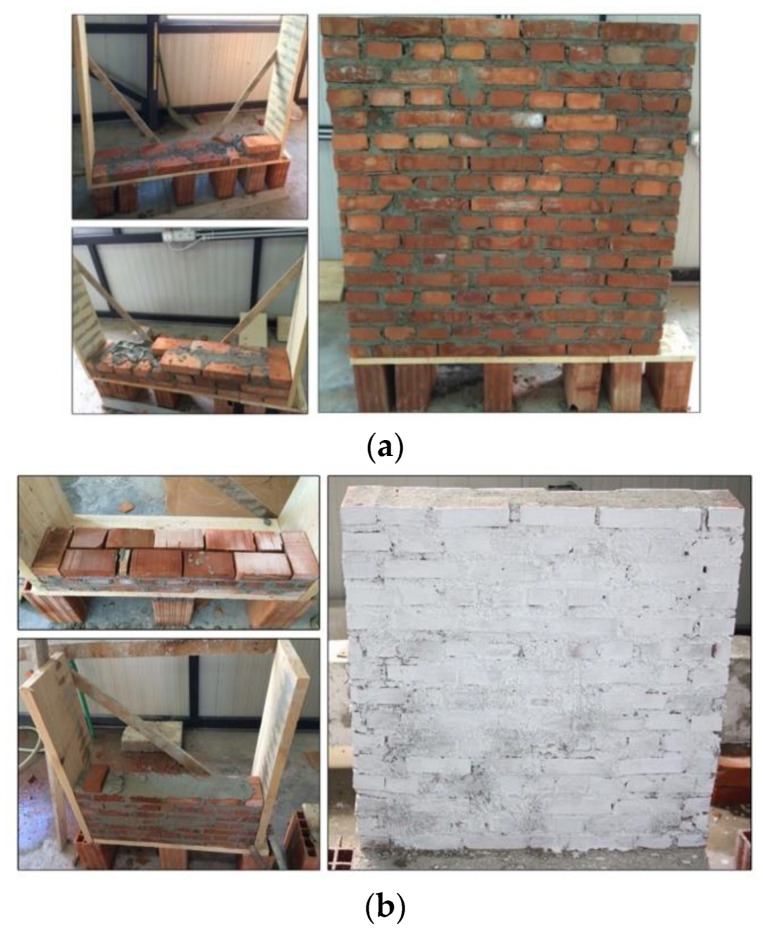
Construction of unreinforced panels: (**a**) full-scale (W-series) and (**b**) half-scale panel (SW-series).

The wall panels were built at the Civil Engineering Laboratory of Epoka University by an experienced brickwork masonry worker using the English bond pattern: only one course of stretchers was applied over a course of headers, i.e., this bond type has two alternating courses of stretchers and headers. The mortar joint thickness for large-scale panels was 15 mm, whereas for the half-scale panels, a 10 mm thickness was used. The walls were left to cure for 28 days. Before testing, a layer of white paint was applied to better visualize the cracks during the tests (Figure 10). 

**Figure 11:**
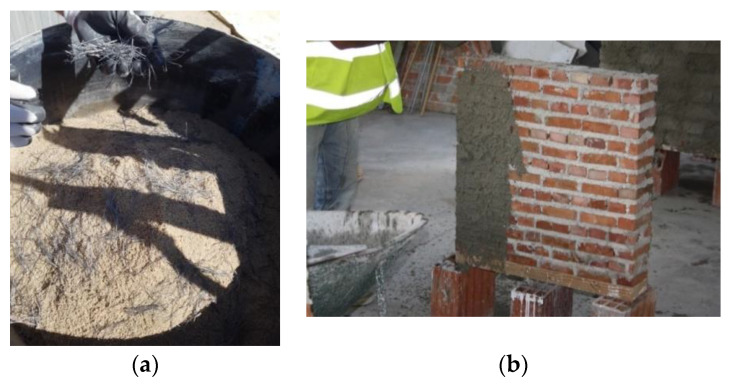
Reinforcement preparation: (**a**) mixing polypropylene fibers with sand and (**b**) application.

**Figure 12:**
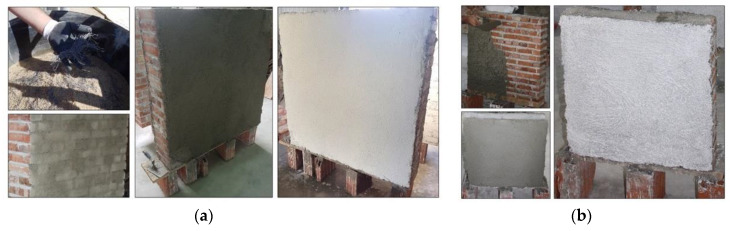
Application of the retrofitting with polypropylene fibers: (**a**) full-scale (W-series) and (**b**) half-scale panel (SW-series).

Twenty-eight days after construction, the wall panels were reinforced with PF. The method consisted of applying a plastering layer that was 25 mm thick (full-size panels) or 15 mm thick (small panels) with a mortar mix of cement:sand volumetric ratio of 1:4, with a water:cement ratio of 0.4 plus 2% of fibers in volume. The preparation of the mix was done by dry mixing PF with sand and cement; then, by adding the water, a plater mix with medium workability was produced. After reinforcement, the panels were left to cure for another 28 days. Similar to the URM panels, a layer of white paint was applied to better visualize the cracks (Figure 11 and Figure 12).

**Figure 13:**
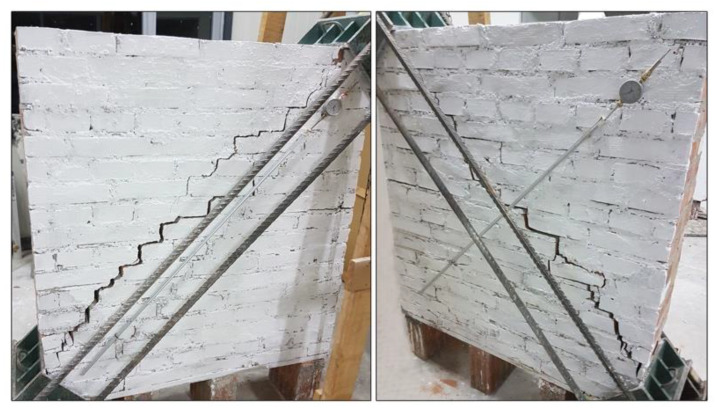
Failure mode of wall panel W-1-URM.

**Figure 14:**
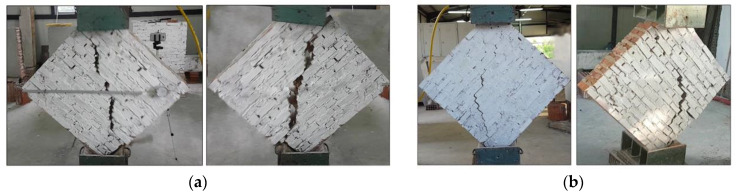
Failure mode of the half-size URM wall panels: (**a**) SW-1-URM and (**b**) SW-2-URM.

After the tests, it was observed that the overall failure mode of the URM panels could be categorized as shear (in-plane) failure; a step-like crack developed along the loaded diagonal through the mortar joints. The main crack started from the center of the wall panel and propagated along the mortar joints toward the panel’s corners. At the same time, some other cracks appeared parallel to the main crack (Figure 13). Since the bricks had much higher tensile and compressive strengths compared to the mortar, the failure occurred only on the mortar bed and head joints and at the interface between the mortar and bricks. The formation of the shear crack, i.e., the failure, was sudden, and all URM panels exhibited a very brittle behavior. The failure mode was similar for both URM full-scale and small panels: in the half-scale panels, the crack and gravity caused a splitting of the wall panel into two halves (Figure 14).

**Figure 15:**
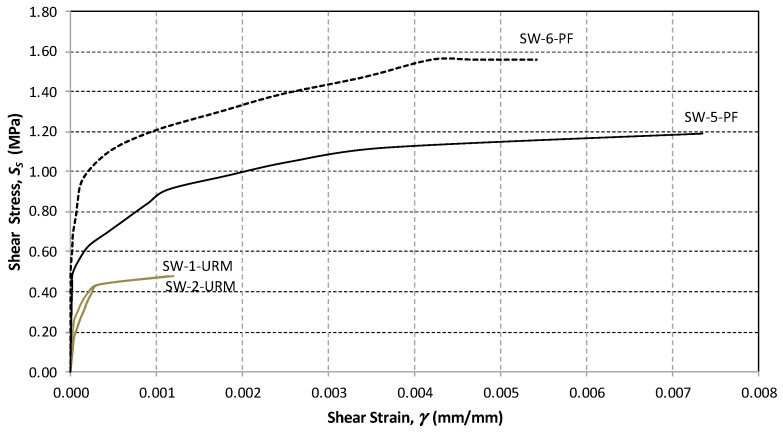
Shear stress versus shear strain plot (half-size panels).

**Figure 16:**
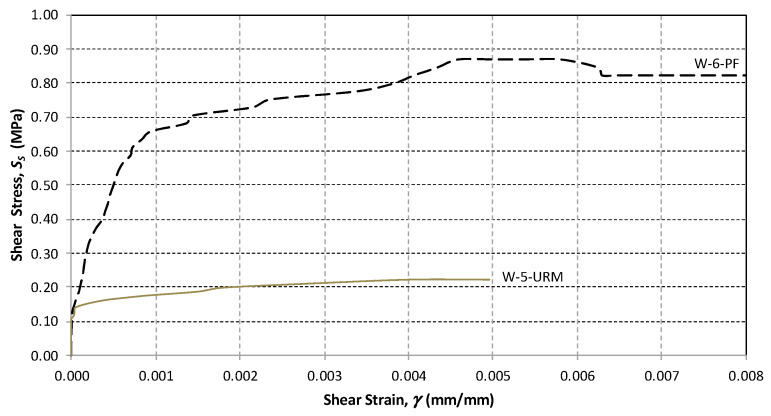
Shear stress versus shear strain plot (large-size panels).

The shear stress versus shear strain response of all panels was determined by calculating the shear stress and the angular strain using Equations (1) and (3), respectively. For the URM panels, the relationship was approximately linear before the crack initiation, followed by a nonlinear response up to the maximum shear capacity (Figure 15 and Figure 16). 

The unreinforced panels exhibited little deformation before the sudden drop in their resistance, thus losing almost all of the load-carrying capacity. This behavior was similar for both types of tested panels (half- and full-size). 

For the in-plane load capacity, the URM half-size panels (SW-1-URM and SW-2-URM) failed at an average maximum load of 54.85 kN, corresponding to a shear strength of 0.497 MPa and an ultimate drift of 0.118%, which represents very brittle behavior (Table 5). The full-size URM panel, namely W-5-URM, displayed similar behavior, but with a lower shear strength. The shear load capacity was higher at 94.66 kN and the corresponding shear strength was only 0.223 MPa. 

**Figure 17:**
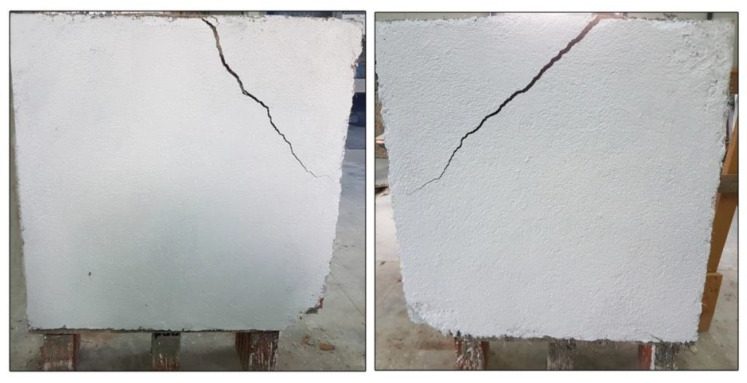
Failure mode of W-6-PF.

The full-size panel reinforced with PF, namely W-6-PF, had a diagonal crack in the upper portion of the wall, starting at the middle of the upper side and ending at the middle of the left side at a clear 45° (Figure 17). This type of failure was not very common in other experimental studies conducted by the authors with TRM reinforcements, where the failure had occurred due to a crack along the loaded diagonal or debonding between masonry and the reinforcement [43].

**Figure 18:**
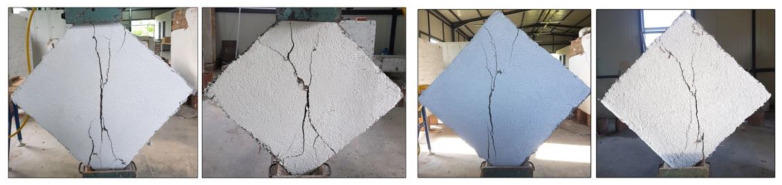
Failure mode of SW-3-PF.

Additionally, it was also observed that the full-scale panel was more ductile than in previous studies, probably due to the different mix of the mortar coating [44,45]. However, the half-size panels had a similar failure mode: a deep vertical crack along the compressed diagonal followed by minor cracks parallel to it (Figure 18). 

For comparison purposes, some of the graphs are plotted to a scale of a maximum angular strain of 0.008. For the polypropylene-reinforced panels, the full-size wall, namely W-6-PF, exhibited a lower shear strength and a lower deformation capacity when compared to the half-size panels. SW-3-PF and SW-4-PF had similar behavior (Figure 15 and Figure 16).

**Figure 19:**
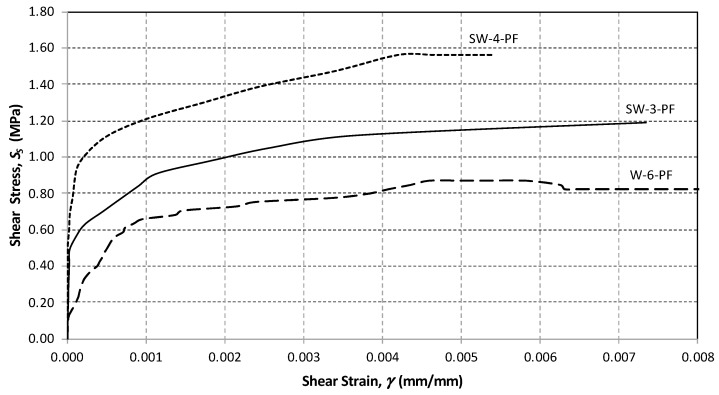
Shear stress versus shear strain plot (wall panels reinforced with polypropylene fibers).

The panels SW-3-PF and SW-4-PF reinforced with polypropylene failed at loads 169.4 and 179.3 kN, respectively. The average shear strength was 1.518 MPa and the ultimate drift 0.418%. W-2-PF failed at 249.1 kN, achieved a shear resistance of 0.587 MPa, and an ultimate drift of 0.434% (Figure 19). 

Finally, the values of *G*_1_ are of particular interest: the shear modulus *G*_1_, calculated in the initial, more elastic phase of the in-plane shear loading, increased dramatically after the application of the PF reinforcement. Table 5 shows that shear modulus *G*_1_ of half-scale panels shifted from 3.96 GPa (URM panels) to 71.52 GPa (PF-reinforced panels). This increase was likely the consequence of the use of a cementitious plastering, which is typically very stiff and the addition of the PFs provided the needed tensile strength to the cementitious mix. The results for the full-scale panels were incomplete for this mechanical parameter. 

From the test results, it was shown that in the half-scale panels, the effect of the application of PF increased the wall shear capacity by 3.17 times and the deformation capacity by 3.54 times, whereas for the full-scale panels, the improvement of the shear-capacity was 2.63 times. 

The reader should be alerted to the limitations of these initial experimental results given the small number of tests carried out in the laboratory. This makes conclusions difficult, especially considering that masonry is highly variable and a difference in the control and treatment can be attributed to this variability and not necessarily to the treatment. However, the general trend seems quite clear: the use of PFs embedded into a cementitious matrix (plaster) produced significant increases in both the shear stiffness and strength compared to unreinforced brickwork masonry.

## 4. Tests Using Polypropylene Nets

The second part of the experimental work involved the use of a PN as a retrofitting method for shear walls. The net had a spacing of 30 mm × 45 mm, and because of its large dimensions, it was decided to only use it on large, full-scale wall panels (LW). Three 1800 mm × 900 mm wall panels were assembled at the Structures Laboratory of the University of Perugia: one was made of a brickwork masonry (nominal thickness 250 mm) and the remaining two were made of rubble stonework (nominal thickness 500 mm). The stone wall panels were only used as an initial attempt to assess the effect of the polypropylene reinforcement on a very common type of masonry used in historic constructions. 

Each wall’s face was reinforced with a PN. The PN was embedded into a cementitious mortar to create a PN-reinforced mortar jacket. The mechanical characteristics of the PN, produced by the Italian company Tenax (Lecco, Italy), are given in Table 2.

**Figure 20:**
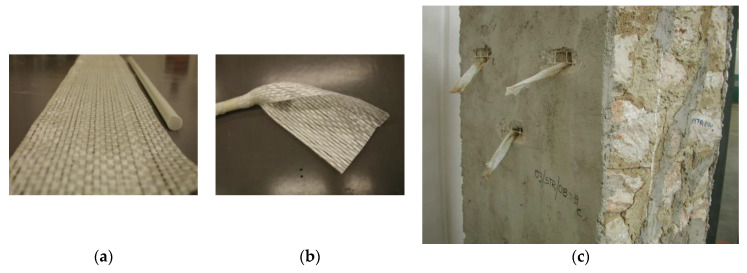
GFRP through-bars were used to connect the two polypropylene jackets. The GFRP bars were wrapped using a fiberglass sheet. This was epoxy-bonded to the polypropylene net: (**a**) GFRP bar and fiberglass sheet, (**b**) wrapping, and (**c**) insertion into the through-holes.

To mutually connect the two PN-reinforced mortar coatings, through-holes were drilled for 12 mm diameter GFRP (glass-fiber-reinforced polymer) rebars (Figure 20a) at a density of 3–4 holes/m^2^. GFRP rebars were wrapped with a GFRP sheet (Figure 20b) and fixed to the PN using an epoxy resin, as shown in Figure 20c. The mortar coating was applied by hand to a thickness of 15 and 25 mm for the brick and stone panels, respectively.

The three wall panels were tested in shear (shear compression test) up to failure using the same procedure for all three panels. However, to evaluate the Young’s modulus, the panels were initially tested in compression by gradually applying a vertical compressive stress of 0.2 MPa. Given the low value of the applied compressive stress, this initial non-destructive test did not damage the walls. The Young’s modulus was calculated based on a normal stress–strain plot using the slope of the secant line between the point in the origin (zero stress, zero strain) and the maximum compressive stress (0.2 MPa) and corresponding strain value.

**Figure 21:**
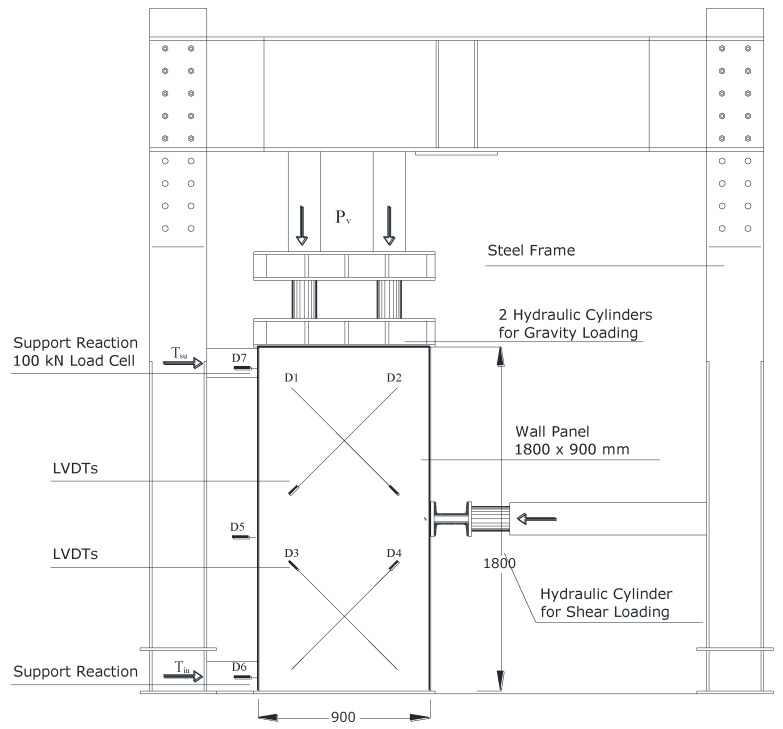
Shear compression test setup (dimensions in mm). LVDTs: Linear Variable Differential Transformers.

It is worth noting that the 1800 mm × 900 mm panel can be seen as two 900 mm × 900 mm semi-panels, one on top of the other. Figure 21 provides further details of the test setup, position of the hydraulic cylinders used for loading, and contact instrumentation (LVDTs: linear variable differential transformers). Before the application of the horizontal shear load, the wall panel was loaded with a vertical compressive load *P_v_*, up to fixed preset value (about 90 kN). The corresponding stress value *σ*_0_ was calculated using:

(6) $$\sigma_{0}=\frac{P_{v}}{A},$$

where *A* is the area of the horizontal cross-section of the panel (900 mm × 250 mm and 900 mm × 500 mm for the brick and stonework panels, respectively).

The maximum shear stress *τ_u_* was calculated from the maximum shear load *T_iu_*, which was measured using a load cell applied on the 900 mm × 900 mm semi-panels:

(7) $$\tau_{u}=\frac{T_{iu}}{A}.$$

The principal tensile stress *σ_I_* is given by:

(8) $$\sigma_{I}=\sigma_{0}\left[-\frac{1}{2}+\sqrt{{\left(b\frac{\tau_{u}}{\sigma_{0}}\right)}^{2}+\frac{1}{4}}\right],$$

where *b* is a shape factor that takes into account the variability of the shear stresses on the horizontal section of the wall. This parameter is assumed by the Italian Building Code [46] and the well-known POR (Pushover Response) method [47] to be equal to 1.5.

The shear strength can be calculated using:

(9) $$\tau_{0}=\frac{\sigma_{I}}{b}.$$

**Figure 22:**
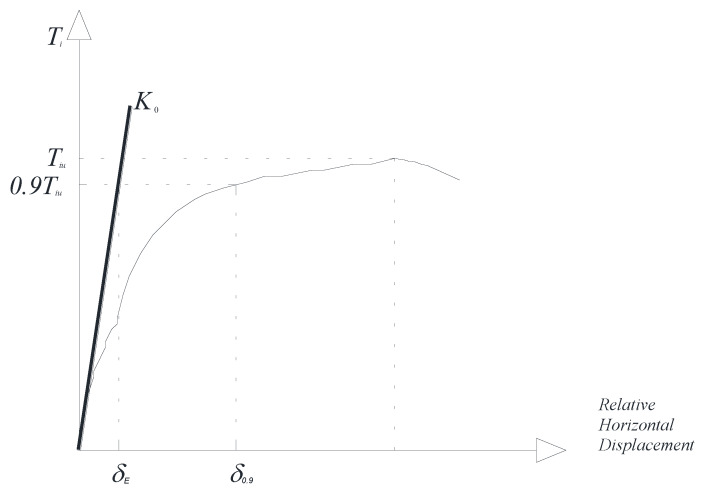
Method used for the calculation of $\delta_{E}$.

It was also decided to measure the shear modulus of the masonry using the available test results. The shear modulus *G* was calculated using the Sheppard interpretation [48,49,50], assuming that the lower 900 mm × 900 mm semi-panel, which is normally the most highly stressed, behaved as an elastic beam perfectly constrained at the bottom. The different levels of constraints of the two semi-panels caused a lack of symmetry in the shear distribution, which was taken into account during the interpretation of the data. The shear modulus *G* was calculated using Equation (9):

(10) $$\frac{1}{K_{0}}=\frac{\delta_{E}}{0.9T_{iu}}=\frac{1.2h}{GA}\left[1+\frac{G}{1.2E}{\left(\frac{h}{t}\right)}^{2}\right],$$

where *t* and *h* are the thickness and height of the masonry panel, respectively; *E* is the Young’s modulus, and *δ_E_* is the relative horizontal displacement between the bottom and middle points of the panel; it was calculated as indicated in Figure 22.

**Figure 23:**
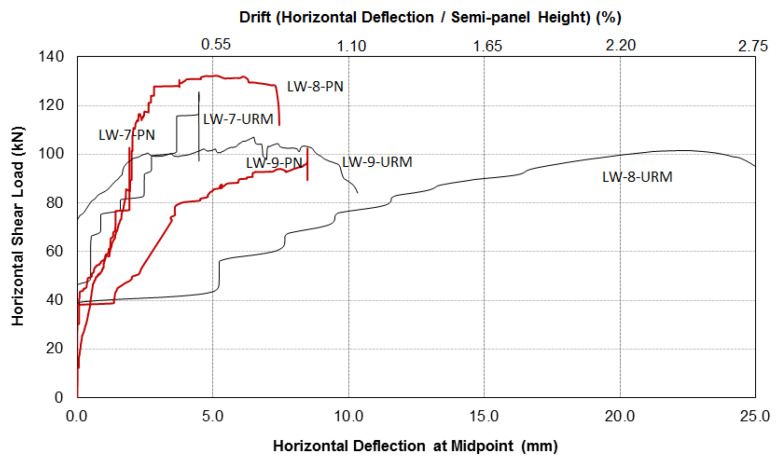
Total shear load versus horizontal deflection/drift (black lines: URM panels, red lines: repaired with PN).

**Figure 24:**
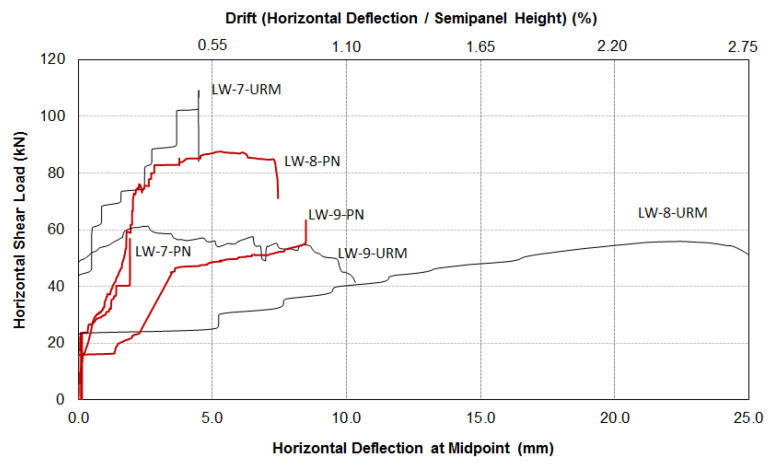
Shear load versus horizontal deflection/drift (black lines: URM panels, red lines: repaired with PN); the graphs are plotted using the shear loads of the semi-panels where failure occurred.

It is worth highlighting that PN was used here as a repair method for cracked walls. The three wall panels were initially tested in the unreinforced configuration (URM) up to failure and were subsequently repaired using the method described above and re-tested. A total of six shear tests were carried out. For the sake of completeness, the data given in Table 6 and Table 7 are partially reproduced and integrated with data given in Table 3. For each of the six panel tests, graphs of shear horizontal load versus the mid-span horizontal deflection have been drawn for both the unreinforced and repaired specimens. These are presented in Figure 23 and Figure 24. The overall conclusion of this experimental work is that in-plane load capacity was not increased using PN. 

If the total shear load was used for the analysis (Table 6 and Figure 23), it can be noted that the stonework URM walls failed at an average shear load of 113.6 kN, and after repair, at 117.5 kN. The PN repair was not able to increase the load capacity but only restored the original capacity of the unreinforced masonry. A similar result can be noted for the brickwork panel (shear capacity tests LW-9-URM: 107.1 kN and LW-9-PN: 102.4 kN). The test results remained similar if the shear capacities in the semi-panel, where the failure was recorded, were more properly used to assess the effectiveness of the PN repair (bold numbers in Table 6). The stone semi-panels exhibited a load capacity of 82.6 kN, while repaired ones had a load capacity of 72.2 kN. For the brick panels, the load-capacity of LW-9-URM was 61.3 kN, and for LW-9-PN, it was 63.4 kN.

**Figure 25:**
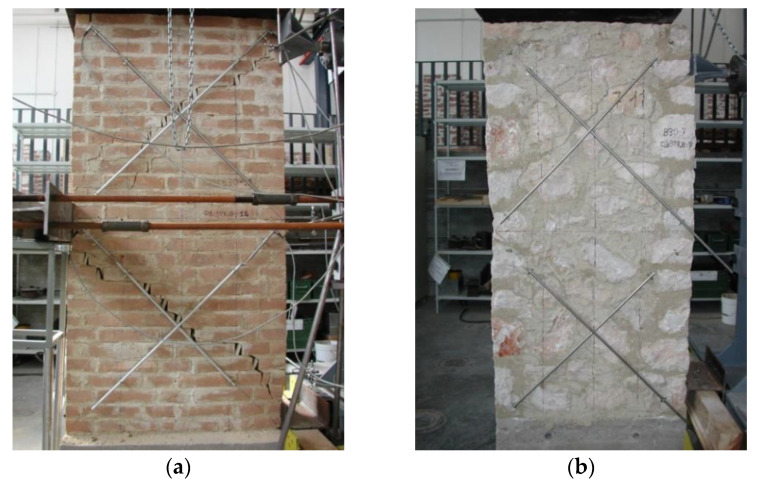
Typical failure mode of the URM wall panels: (**a**) brickwork panel and (**b**) stonework panel.

The failure mode of the URM walls was due to the formation of stepped diagonal cracks in one or both semi-panels. For brickwork masonry, these cracks usually appeared along the mortar joints between the brickwork and followed the mortar joints diagonally upward in a staircase pattern (Figure 25a). The cracks typically first appeared in the lower semi-panel as a consequence of the cited lack of symmetry in terms of the semi-panel’s constraints. For stonework panels, the cracks had a more diagonal orientation (diagonal crack at 45° along the semi-panel’s diagonal in compression). The shear capacity was clearly governed by the mechanical characteristics of the mortar used for construction and its bonding properties with the stone and the bricks. It seems that the mechanical properties of the stone and bricks had little influence on the structural response of the shear walls.

Failure occurred when the loading produced diagonal cracks in the lowest half-panel and high bond stresses occurred between the polypropylene fabric and the masonry substrate, causing delamination of the reinforcement and the collapse of the panel. The delamination occurred because of poor bonding between FRP and the masonry surface due to the use of hydraulic mortar.

The failure mode of all repaired wall panels (test labels LW-7-PN, LW-8-PN, and LW-9-PN) was similar: for a low level of the shear load, the bonding between the PN-reinforced mortar jacket and the masonry substrate was functional. This caused an interesting increase in the shear stiffness of the walls, as demonstrated by the increase of the shear modulus values compared to the URM panels (Table 7). During this initial phase of the loading, the application of the PN-reinforced jacket was ultimately effective. 

**Figure 26:**
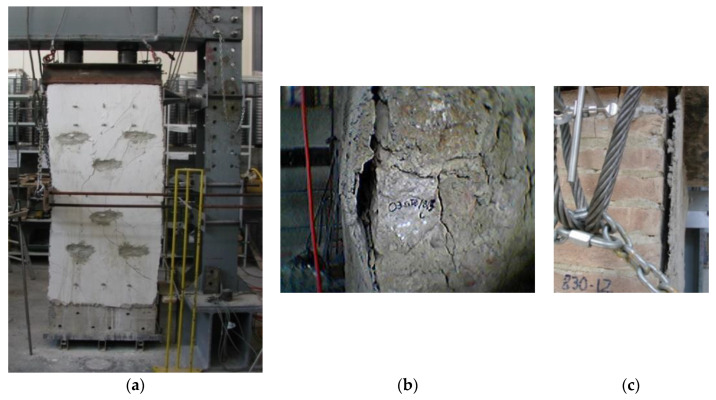
Typical failure mode of repaired wall panels: (**a**) stonework panel, (**b**) detail of the failure mode (stonework panel), (**c**) brickwork panel.

Because the wall panels were already cracked along their diagonals, the PN acted to stitch the cracks, but the very different deformation capacity of the PN (very high deformation capacity, low Young’s modulus) and the mortar used for the jacket (very low deformation capacity, high Young’s modulus) meant that the tensile stresses in the region of the shear cracks were essentially absorbed by the more stiff mortar. Extensive cracks re-opened in the masonry and also formed in the mortar jacket (Figure 26a). By increasing the shear load, the PN-reinforced jacket extensively detached from the masonry for both the stonework and brickwork (Figure 26b,c, respectively).

The “incompatibility” between the PN and mortar jacket in terms of mechanical properties was the major reason for this unsatisfactory behavior of the repaired walls. To overcome this problem, high-deformation mortars could be used (for example aerial lime-based), but this could further compromise the masonry-to-jacket and jacket-to-PN bonding.

## 5. Conclusions

In this paper, the test results of an experimental investigation of stone and brickwork shear walls that were strengthened with various types of polypropylene have been presented. Polypropylene short fibers and polypropylene nets were used to reinforce or repair pre-damaged shear walls. The study was essentially the first phase of a research program intended to investigate the use of polypropylene in the strengthening of old masonry walls against the destructive action of earthquakes.

Based on the test results, the following conclusions were drawn:

- The use of polypropylene fibers, embedded into a cementitious matrix to reinforce brickwork walls was ultimately effective. The maximum increase in the in-plane load capacity was achieved when two jacket coatings were used as a retrofitting method: for the half-scale and full-scale panels, the shear capacity increases were 317% and 263%, respectively, compared to the unreinforced wall panels.
- The repair of cracked wall panels with polypropylene nets was ineffective. For both the brick and stonework shear walls, the strength and ductility of the repaired wall panels did not increase after repair. This was likely the consequence of the low Young’s modulus of the polypropylene net and its low bonding properties. The PN-reinforced mortar jacket detached from the masonry substrate during shear loading.
- Both the URM and polypropylene-retrofitted wall panels showed distinct bi-linear behavior for low and high in-plane loads, and the slope of the second linear part of the curve only depended on the loading procedure and the progressive development of the shear cracks, and not on the effect of the polypropylene reinforcement or repair. The stress–strain response of the retrofitted walls was similar to that of URM walls and lacked any distinct post-peak strain-hardening behavior.

It was anticipated that polypropylene nets were particularly susceptible to problems of deboning from the masonry substrate and also from the mortar jacket due to problems of “incompatibility” between these materials. Further research must be conducted in this area, both to increase the bonding properties and to reduce the incompatibility, mainly in terms of the deformation capacity.

## Figures and Tables

**Table 1:** Technical specifications of polypropylene fibers (100% polypropylene as a chemical base) used for the reinforcement of shear walls (from the producer’s datasheet).

Property	Value/Rating
Specific gravity (g/cm³)	0.91
Fiber length (mm)	12
Fiber diameter (μm)	18
Melt point (°C)	160
Ignition point (°C)	365
Thermal conductivity	Low
Electrical conductivity	Low
Specific surface area of the fiber (m^2^/kg)	250
Acid resistance	High
Alkali resistance (%)	100
Tensile strength (MPa)	300–400
Young’s modulus (MPa)	4000

**Table 2:** Technical specifications of a polypropylene net (100% polypropylene as a chemical base) used for the repair of shear walls (from the producer’s datasheet).

Property	Shape/Value
Mesh shape	Quadrangular
Mesh size (mm)	30 × 45
Net mesh size (mm)	27 × 42
Weight density (kg/m^2^)	0.16
Tensile strength—warp (kN/m)	9.3
Elongation at failure—warp (%)	16
Tensile strength—weft (kN/m)	17
Elongation at failure—weft (%)	13
Young’s modulus—polypropylene (MPa)	1450

**Table 3:** Test matrix.

Test Label	Nominal Wall Dimensions			Reinforcement Type
	Height *h* (mm)	Width *w* (mm)	Thickness *t* (mm)	
SW-1-URM	650	650	120	Unreinforced
SW-2-URM	650	650	120	Unreinforced
SW-3-PF	650	650	120	Polypropylene fibers
SW-4-PF	650	650	120	Polypropylene fibers
W-5-URM	1200	1200	250	Unreinforced
W-6-PF	1200	1200	250	Polypropylene fibers
LW-7-URM	1800	900	500	Unreinforced
LW-8-URM	1800	900	500	Unreinforced
LW-9-URM	1800	900	250	Unreinforced
LW-7-PN	1800	900	500	Polypropylene net
LW-8-PN	1800	900	500	Polypropylene net
LW-9-PN	1800	900	250	Polypropylene net

**Table 4:** Results of the material characterization tests.

Specimen	Water Absorption (%)	Compressive Strength (MPa)	Tensile Strength (MPa)
Full-dimension brick	22 (0.13)	24.30 (0.12)	4.54 (0.11)
Half-size brick	17 (0.04)	20.13 (0.10)	3.82 (0.16)
Mortar (wall construction)	–	3.02 (0.10)	1.97 (0.02)
Mortar (plastering)	–	17.64 (0.17)	2.12 (0.09)
Brick masonry (half bricks, 10 mm bed joints)	–	13.69 (0.08)	–
(full bricks, 15 mm bed joints)	–	8.36 (0.11)	–

Coefficient of variation values are found in the brackets.

**Table 5:** Results of the shear tests (diagonal compression tests).

Panel Label	Maximum In-Plane Load, P (kN)	Shear Strength, *S_S_* (MPa)	Ultimate Drift, *δ* (%)	Shear Modulus, *G*_1_ (GPa)	Shear Modulus, *G*_2_ (GPa)
SW-1-URM	54.80	0.496	0.122	5.57	2.567
SW-2-URM	54.90	0.498	0.113	2.34	1.828
SW-URM-average	54.85	0.497	0.118	3.96	2.198
SW-3-PF	169.4	1.535	0.367	26.01	1.069
SW-4-PF	179.3	1.626	0.468	117.05	1.852
SW-PF-average	174.4	1.580	0.418	71.53	1.461
W-5-URM	94.66	0.223	0.280	3.91	0.762
W-6-PF	229.1	0.587	0.434	N/A	0.182

**Table 6:** Results of shear tests (LW panels); the results are in terms of the in-plane load capacities.

Test Label	Panel Dimensions (cm)	Compressive Vertical Load^+^ *P_v_* (kN)	Maximum Shear Load (kN)	Maximum Shear Load (kN) (Upper Semi-Panel)	Maximum Shear Load (kN) (Lower Semi-Panel)
LW-7-URM *	90 × 48.6 × 180.5	93.1	125.6	**109.3**	16.6
LW-8-URM *	90 × 48.6 × 190	80.7	101.6	45.7	**55.9**
LW-9-URM **	90 × 25 × 179	89.2	107.1	50.0	**61.3**
LW-7-PN *	90 × 54.5 × 180.5	90.1	102.7	46.0	**56.8**
LW-8-PN *	90 × 52 × 190	86.6	132.3	52.0	**87.6**
LW-9-PN **	90 × 26 × 180	84.5	102.4	**63.4**	43.4

* Stonework masonry, ** brickwork. The capacity values in bold identify the semi-panels where failure occurred.

**Table 7:** Results of shear tests (LW panels); the results are in terms of the stresses.

Test No.	Panel Dimensions (cm)	Shear Load at Failure *T_iu_* (kN)	Shear Stress *τ_u_* (MPa)	Compressive Stress *σ*_0_ (MPa)	Shear Strength *τ*_0_ (MPa)	Shear Modulus *G* (MPa)
LW-7-URM *	90 × 48.6 × 180.5	109.3	0.250	0.213	0.189	77
LW-8-URM *	90 × 48.6 × 190	55.9	0.128	0.184	0.080	133
LW-9-URM **	90 × 25 × 179	61.3	0.272	0.396	0.171	100
LW-7-PN *	90 × 54.5 × 180.5	56.8	0.130	0.206	0.078	309
LW-8-PN *	90 × 52 × 190	87.6	0.200	0.198	0.145	343
LW-9-PN **	90 × 26 × 180	63.4	0.282	0.376	0.183	234

* Stonework masonry, ** brickwork.
